# A quantitative exploration of health care workers opinions and attitudes towards HIV-infected co-workers and patients in Beijing, China

**DOI:** 10.1186/1471-2334-14-S2-P12

**Published:** 2014-05-23

**Authors:** Xiaona Liu, Xinying Sun, Lenneke van Genugten, Yuhui Shi, Yanling Wang, Wenyi Niu, Jan Hendrik Richardus

**Affiliations:** 1 Erasmus MC, University Medical Center of Rotterdam, Rotterdam, the Netherlands

## Introduction

Health care workers (HCWs) face moral and practice-related dilemmas when working with co-workers and patients with HIV. This study aims to understand the underlying stigmatizing opinions and attitudes of HCWs that may drive discrimination towards HIV-infected co-workers and patients in the workplace.

## Materials and methods

A cross-section survey was conducted among 392 HCWs (113 doctors, 236 nurses and 43 technicians) in two tertiary hospitals in Beijing between May and July 2010. Participants were asked by a self-administered anonymous written questionnaire about their socio-demographic characteristics; experience on HIV-related training, testing, and percutaneous injury; and the extent of their agreement with 9 rigid opinions regarding managing HIV-infected co-workers, and with 10 negative attitudes regarding working with HIV-infected patients on a 5-point Likert scale. Pearson correlations and multivariate regression analyses were performed using SPSS 20.0 to study possible correlates of the general opinion and attitude.

## Results

Participants perceived a high risk of HIV transmission in both co-worker and HCW-patient relationships (86% and 87% respectively). Half of participants agreed that HCWs should routinely and mandatorily receive HIV-tests, HCWs with HIV should disclose their diagnosis to relevant parties, and should be restricted from performing invasive procedures. Participants had a negative feeling towards patients infected through sexual contact (80%), and believed that HCWs have the right to refuse to care for infected patients (51.8%), and that those patients should be treated only in designated hospitals (87.0%). Almost all HCWs intended more or less to avoid performing invasive clinical procedures or nursing services for HIV-infected patients (91.0%). After corrected by other factors (e.g., age, gender), nurses retain significantly more stigmatizing attitudes towards HIV-infected patients than doctors (β = -0.19, 95%CI: -0.32, -0.05) and technicians (β = -0.15, 95%CI: -0.32, 0.02).

## Conclusions

The identified rigid opinions on managing HIV-infected co-workers, together with stigmatizing attitudes towards HIV-infected patients, underscores an urgent need to decrease stigmatizing attitudes and prevent discriminatory practices in health care settings. Rectifying gaps in ethics, legislation, and practice is critical to reach the balance between ensuring rights of people with HIV and preventing HIV transmission in the workplace.

